# Regulation of Chromatin Accessibility by the Farnesoid X Receptor Is Essential for Circadian and Bile Acid Homeostasis In Vivo

**DOI:** 10.3390/cancers14246191

**Published:** 2022-12-15

**Authors:** Haider M. Hassan, Oladapo Onabote, Majdina Isovic, Daniel T. Passos, Frederick A. Dick, Joseph Torchia

**Affiliations:** 1 Department of Biochemistry, Western University, London, ON N6A 5C1, Canada; 2 Department of Oncology, London Regional Cancer Program and the Lawson Health Research Institute, London, ON N6A 5W9, Canada; 3 Department of Pathology and Laboratory Medicine, Western University, London, ON N6A 5C1, Canada

**Keywords:** Farnesoid X Receptor, chromatin accessibility, transcription factor, CRISPR/Cas9, liver cancer, ATAC-seq

## Abstract

**Simple Summary:**

The Farnesoid X Receptor (FXR) is a nuclear receptor that regulates the expression of several genes involved in the metabolism of bile acids (BAs) which is essential for normal liver function. In this study, we used the CRISPR/Cas9 system to generate a novel FXR knockout mouse model and demonstrate that FXR deletion in hepatocytes is associated with a global reduction in chromatin accessibility. The loss of chromatin accessibility was predominantly localized to promoter-associated transcription factor motifs, such as the NFγ/CBP and the KLF/SP1 family of pioneer transcription factors. Importantly, we demonstrate that the loss of FXR mediated chromatin accessibility contributes to dysregulation in bile acid and circadian homeostasis in hepatocytes.

**Abstract:**

The Farnesoid X Receptor (FXR) belongs to the nuclear receptor superfamily and is an essential bile acid (BA) receptor that regulates the expression of genes involved in the metabolism of BAs. FXR protects the liver from BA overload, which is a major etiology of hepatocellular carcinoma. Herein, we investigated the changes in gene expression and chromatin accessibility in hepatocytes by performing RNA-seq in combination with the Assay for Transposase-Accessible Chromatin with high-throughput sequencing (ATAC-seq) using a novel FXR knockout mouse model (*Fxr^ex5Δ^: Nr1h4^ex5Δ/ex5Δ^*) generated through CRISPR/Cas9. Consistent with previous *Fxr* knockout models, we found that *Fxr^ex5Δ^* mice develop late-onset HCC associated with increased serum and hepatic BAs. FXR deletion was associated with a dramatic loss of chromatin accessibility, primarily at promoter-associated transcription factor binding sites. Importantly, several genes involved in BA biosynthesis and circadian rhythm were downregulated following loss of FXR, also displayed reduced chromatin accessibility at their promoter regions. Altogether, these findings suggest that FXR helps to maintain a transcriptionally active state by regulating chromatin accessibility through its binding and recruitment of transcription factors and coactivators.

## 1. Introduction

Hepatocellular carcinoma (HCC) is the most common primary liver malignancy and the third leading cause of cancer-related mortalities worldwide [1]. The high mortality rate of HCC is partly attributed to the numerous etiologies associated with the disease and the extensive heterogeneity observed in HCC tumors [2]. Chronic viral hepatitis and alcohol consumption are the most important risk factors for HCC development worldwide [3]. Nevertheless, metabolic disorders such as obesity, type 2 diabetes (T2D), and non-alcoholic fatty liver disease (NAFLD) are rapidly becoming the leading causes of HCC in developed nations [4]. NAFLD is characterized by hepatic steatosis, insulin resistance, and chronic inflammation, all of which contribute to progression of NAFLD to end-stage liver diseases such as non-alcoholic steatohepatitis (NASH), advanced fibrosis, cirrhosis, and HCC [5].

The dysregulation of bile acid (BA) homeostasis is a major driving force of NAFLD development and progression [6,7]. BAs primarily function as physiological detergents that facilitate the intestinal absorption of lipids and fat-soluble vitamins [8]. They can also function as signaling hormones that regulate their own synthesis via negative feedback inhibition involving the Farnesoid X Receptor (FXR, also referred to as FXRα or *Nr1h4*) signaling [9]. FXR is a BA receptor that belongs to the nuclear receptor superfamily of ligand-activated transcription factors [10,11,12]. FXR is highly expressed in the liver and intestine, where it functions as a BA sensor that regulates the expression of genes involved in lipid, glucose, and bile acid (BA) metabolism [13]. The maintenance of physiological BA concentrations within the liver is achieved through the combined actions of hepatic Small Heterodimeric Protein (*Shp*/*Nr0b2*) and the endocrine hormone FGF 15/19, both of which are directly regulated by FXR. In the liver, BA-dependent activation of FXR induces expression of *Nr0b2*, an atypical nuclear receptor that lacks a DNA binding domain. *Nr0b2* acts as a corepressor for the nuclear receptors HNF4α and LRH-1 which in turn repress the transcription of *Cyp7a1*, the rate-limiting enzyme of the classical BA synthesis pathway [14]. In the intestine, FXR activation promotes the transcription of *Fgf15/19*, which is secreted and circulates to the liver to activate the hepatic FGF receptor 4 (FGF4). The activation of FGF4 represses *Cyp7a1* gene transcription via extracellular regulated protein kinases 1/2 (ERK1/2)/cJun of the mitogen-activated protein kinase (MAPK) pathway [15]. Ultimately, the combined actions of BA-induced activation of FXR in the liver and intestine represses BA synthesis in the liver. This negative feedback response is critical for maintaining BA homeostasis in the gastrointestinal (GI) tract.

At the transcriptional level, FXR regulates gene expression by binding to FXR response elements (FXREs) either as a monomer or a permissive heterodimer with Retinoid X Receptor (RXR) [11,16]. The canonical FXRE contains two copies of a consensus sequence (AGGTCA) arranged as inverted repeats separated by one nucleotide (IR1). In the absence of ligand, FXR is bound in its inactive state to corepressor proteins such as NCOR1 [17,18]. BA binding to the ligand-binding domain of FXR triggers a conformational change which results in dissociation of the corepressor complex and association with coactivator proteins such as CBP/p300, SRC-1, and the methyltransferase CARM1 [19,20]. Although the transcriptional changes associated with a loss of FXR have been characterized [21,22], the dynamics of chromatin accessibility associated with alterations in gene expression in hepatocytes remain unclear.

In this study, we used the CRISPR/Cas9 system to generate a novel FXR knockout mouse model (*Fxr^ex5∆^: Nr1H4^ex5∆/ex5∆^*). We utilize the Assay for Transposase Accessible Chromatin sequencing (ATAC-seq) in combination with RNAseq to examine the consequences of loss of FXR on chromatin accessibility and its relationship to changes in gene expression, and transcription factor binding in hepatocytes. We find that the *Fxr^ex5∆^* mice develop a late-onset HCC with complete penetrance, as well as elevated serum and hepatic bile acids. Mechanistically, we demonstrate that FXR deletion is associated with a dramatic loss of chromatin accessibility at specific transcription factor binding sites, primarily at the promoter region of target genes. We show that FXR binds within the promoter or distal intergenic regions at its target genes and that the deletion of FXR is associated with loss of chromatin accessibility resulting in transcriptional dysregulation of its target genes. Collectively, these findings provide key insights into the transcriptional dynamics associated with FXR regulation of genes.

## 2. Materials and Methods

### 2.1. Generation of the Fxr^ex5∆^ Mice

All mouse experiments were done in compliance with the Institutional Animal Care and Use Committee guidelines at London Regional Cancer Center at Western University. *Fxr*-null mice (*Fxr^ex5∆^: Nr1H4^ex5∆/ex5∆^*) were generated using the CRISPR/Cas9 genome editing by the London Regional Transgenic and Gene Targeting facility. Briefly, zygotes generated from in vitro fertilization of C57BL/6NCrl oocytes were microinjected with Cas9 mRNA (TriLink Biotechnologies, San Diego, CA, USA) and an exon 5 directing gRNA (Table 1). into the pronucleus. All injected zygotes were incubated overnight at 37 °C, and all embryos that developed to the 2-cell stage were implanted into 0.5 dpc pseudopregnant CD-1 females the following morning. Founders were bred with wildtype C57BL/6N mice to generate heterozygous mice. Heterozygous mice were intercrossed to generate homozygous mutants for characterization of mutant alleles. The primers used for genotyping are listed in Table 2. The described 47bp deletion allele was selected because it is deficient for FXR protein expression. This allele is formally called *Nr1h4^ex5∆^* but we refer to it as *Fxr^ex5∆^* for simplicity in this report. Livers were harvested from 3-week-old *Fxr*-null mice and wildtype mice for gene expression analysis and protein expression analysis.

### 2.2. Hepatocyte Isolation and Cryopreservation

The hepatocyte isolation protocol was performed as previously described [23]. Briefly, 8-week-old mice were anesthetized using avertin (20 mg/mL) prior to placement on the dissection tray. We chose this timepoint because cannulation of older mice is more challenging due to the increased fat lining around the vena cava. A “U”-shaped incision was made through the skin, and the intestines were moved to the left to reveal the portal vein and vena cava. Then, a catheter was injected into the vena cava. The portal vein was snipped, followed by perfusion of HBSS (Containing 0.5 mM EDTA) prewarmed to 37 °C for 20 min at a flow rate of 3 mL/min. Then, the liver was digested using collagenase (30 mg in 40 mL low-glucose DMEM) at a flow rate of 2.5 mL/min. The liver was removed and dissociated in a sterile Petri dish under laminar flow by physical scraping or moving back and forth using surgical tweezers until the media turned opaque. The media containing hepatocytes was filtered using a 100 µm, followed by a 70 µm sterile filters, prior to centrifugation at 50× *g* for 2 min at 4 °C—No breaks were used during deceleration. 

The hepatocytes were washed twice with ice-cold low glucose DMEM containing 5% FBS, prior to density gradient centrifugation using Percoll (9 mL Percoll, 1 mL of 10× PBS.). The hepatocytes were further washed using low glucose DMEM containing 5% FBS, prior to resuspension in the cryopreservation media (40 mL low-glucose DMEM, 5 mL DMSO, 5 mL FBS) at a concentration of 1 × 10^6^ cells/mL. A viability of 95% or higher was required for cryopreservation. Hepatocytes were also plated to ensure viability and purity. The hepatocytes for cryopreservation were placed in a cryofreezing container filled with isopropanol according to manufacturer instructions and placed at −80 °C overnight.

### 2.3. Tagmentation and ATACseq

A vial of cryopreserved hepatocytes was removed from −80 °C freezer and immediately placed in a 37 °C water bath for 3 min. A 10 μL aliquot of the hepatocytes was taken to measure viability using trypan blue. A minimum viability of 80% was required prior to proceeding with tagmentation. Immediately after 2 min at 37 °C, the thawed hepatocytes were washed using 10 mL of pre-warmed D-PBS in a 14 mL falcon tube. The hepatocytes were centrifuged at 50× *g* for 3 min at 4 °C with brakes turned off. After removing the supernatant, the hepatocytes were resuspended in 10 mL of ice-cold nuclei isolation buffer (10 mM Tris-HCl, pH 7.5, 2 mM MgCl_2_, 3 mM CaCl_2_, 10% Glycerol) and left on ice for 5 min. Then, the hepatocytes were centrifuged at 400× *g* for 5 min with brakes on. The nuclei were stained with trypan blue, visualized under microscope, and counted using a cell counter. Approximately 200,000 nuclei were used for tagmentation in the Tn5 buffer (50 mM Tris-HCl, pH 7.5, 25 mM MgCl_2_, 50 mM DMF) using 10 µL of Tn5. The tagmentation was performed at 37 °C for 30 min with shaking at 700 rpm. The DNA was immediately isolated using the Zymo DNA clean and concentrator-5 (Cat no. D4013). Then, PCR was performed using the illumina i5 and i7 indexing primers containing adaptors for 8–12 cycles (75 °C, 5 min; 98 °C 30 s; 8–12 cycles of (98 °C 10 s, 63 °C 30 s, 72 °C 30 s); 72 °C 5 min, 4 °C indefinitely). The PCR amplified DNA was cleaned using the Zymo DNA clean and concentrator -5, double size selected using Ampure XP beads (Cat no. A63880) and quantitated using Collibri library quantitation kit (Cat no. A38524100). The libraries were analyzed for fragmentation via Agilent bioanalyzer and sequenced using illumina NovaSeq high throughput sequencer (150 bp × 150 bp paired end; 50 million read pairs per sample).

### 2.4. High Throughput RNA Sequencing

For the RNA-seq, the sample quality was assessed using the Agilent 2100 Bioanalyzer. Qualifying samples were then prepped following the standard protocol for the NEBnext Ultra ii Stranded mRNA (New England Biolabs) at the University of British Columbia. Sequencing was performed on the Illumina NextSeq 500 with Paired End 42 bp 3 42 bp reads, with a depth of 50 million reads per sample. The raw data was aligned to the mm10 mouse genome using the STAR aligner and the gene list was generated using cufflinks. A list of differentially expressed genes was generated using q < 0.05 as the cutoff for significance. The heatmap was generated using the Bioconductor gg-plot addon for the R software and Morpheus from broad institute. For the TCGA analysis, the data was downloaded using the firehose website, and analyzed using the R-software.

### 2.5. RT-PCR

Total RNA from liver tissues was extracted using an RNAzol solution (Sigma, St. Louis, MO, USA) as per the manufacturer’s protocol. The cDNA was synthesized using the Applied Biosystems Reverse Transcription Kit as per manufacturer’s protocol. For RT-PCR, cDNA was PCR amplified using primers from Table 3. Samples were loaded onto a 2% agarose gel and electrophoresed for 30 min at 180 V. After electrophoresis, DNA fragments with visualized using ethidium bromide staining and imaged using the ChemiDoc XRS imaging system (BioRad, Hercules, CA, USA).

### 2.6. Protein Extraction and Western Blot

For protein extraction, whole livers/hepatocytes were homogenized in 1 mL of ice-cold RIPA lysis buffer (150 mM NaCl, 0.5% sodium deoxycholate, 0.1% SDS, 1% NP-40, 50 mM Tris-HCl, pH 8) containing 1× Halt Protease Inhibitor Cocktail (Thermo Scientific, Rockford, IL, USA). Lysates were incubated on ice for 15 min, centrifuged at maximum speed (13,000× *g*) at 4 °C for 15 min and the supernatant was retained. Protein concentrations were determined using the Bradford assay. For Western blot, 50 μg protein samples were loaded onto a 4–12% gradient Bis-Tris NuPAGE gel (Invitrogen, Carlsbad, CA, USA), subjected to SDS-PAGE, and transferred onto a PVDF membrane. PVDF membranes were incubated in blocking buffer consisting of 5% skim milk in PBS for one hour and hybridized overnight with the appropriate primary antibody at the indicated dilution. After five ten-minute washes in blocking buffer, membranes were hybridized for one hour with the appropriate secondary antibodies. The membranes were then washed 5 times in blocking buffer and the blots were developed using the Clarity Western ECL Substrate (Bio-Rad, Hercules, CA, USA) and imaged using the ChemiDoc XRS imaging system (Bio-Rad, Hercules, CA, USA). The primary antibodies were used in this study are listed in Table 4.

### 2.7. Liver Histology

For histology, whole livers were harvested from aging FXR knockout and control mice at endpoint. Liver samples containing either normal or tumor regions were fixed in 10% formalin solution and embedded in paraffin. Sections were cut at 4 μm and stained with hematoxylin and eosin.

### 2.8. Total Bile Acid Analysis

The total bile acid analysis was performed as previously described [24]. Briefly, liver tissue was homogenized in 1 mL of 70% ethanol and then incubated at 50 °C for 2 h. The homogenate was spun down at 10,000 rpm, and the supernatant was dried, resuspended in 200 mL of water, aliquoted and kept at −80 °C until analysis. For the serum BAs, blood was collected from the heart at end-point. Blood was centrifuged at 3000 rpm at 4 °C, and the supernatant was kept at −80 °C until analysis. For the BA analysis, the Total Bile Acid Assay Enzyme Cycling Method Kit (Diazyme, Poway, CA, USA) was used. Bio-Tek Synergy H4 Hybrid reader was used to analyze the samples at 37 °C over a 4 min period, with readings taken every 30 s at 405 nm. The calibration curve was generated by taking the difference between OD405 readings from 30 s and 4 min and correlating to concentration of standard used. Then, 4 μL of the liver ethanolic extract or serum sample was used for analysis and the con- centration of BAs was determined using the standard calibration curve.

### 2.9. Chromatin Immunoprecipitation (ChIP)

The chromatin immunoprecipitation assay was performed as previously described [5]. Briefly, approximately 1 million hepatocytes were washed with ice-cold D-PBS and then incubated with 1% formaldehyde (*v*/*v*) for 10 min with rotation at room temperature. The homogenate was then lysed, sonicated, and incubated with 50 μL of premixed protein A/G DYNABEADS overnight. The DYNABEADS were then washed and the resulting chromatin was eluted using the elution buffer (1% SDS, 0.1 M NaHCO_3_). The eluted chromatin was reverse-crosslinked and the resulting ChIP DNA was analyzed by qPCR using primers outlined in Table 3.

### 2.10. Bioinformatics

STAR and bowtie2 were used for the alignment for RNAseq and ATAC-seq datasets, respectively, to the mm10 genome. Cufflinks was used for differential gene expression analysis. All ChIP-seq data were obtained from the ChIP-atlas database (https://chip-atlas.org (accessed on 24 May 2022)). For ATACseq, the BAM files were sorted, indexed and filtered for maq quality (>30), mitochondrial reads and read duplicates using ChIPseq and ATACseq Processing and Peak calling Software (CAPPS, version 2.1; Link to Github page: https://github.com/HaiderMDev/CAPPS-Processing-and-QC). CAPPS was also used to call peaks using MACS2, generate the fragment size distribution graph via picards, and generate BigWig files. Appropriate fragmentation size distribution was essential for downstream analysis. CAPPS is a two-part data analysis software package with a GUI and is written in python and R. CAPPS streamlines pre-processing and data analysis of ATAC-seq and ChIPseq datasets.

Differential peak analysis between control and *Fxr^ex5∆^* hepatocytes was performed using CAPPS-Data Analysis available in R (Link to Github page: https://github.com/HaiderMDev/CAPPS-Data-Analysis). Homer was used for Motif analysis (findMotifgenome.pl) and for annotating peaks around promoter or distal intergenic regions (annotatepeak.pl). Bedtools, DeepTools and CAPPS-Data Analysis were used to generate heatmap plots, perform data overlaps and for pathways analysis. All peak summit plots were generated using IGV.

All Western blots are representative of at least duplicate experiments. Statistical analyses were performed using GraphPad Prism 5 using the Student’s T test (2 groups), or ANOVA with Tukey post hoc (3 groups or more). All *p* values over 0.05 were deemed not significant. Significance is indicated as follows: *, *p* < 0.05; **, *p* < 0.01; ***, *p* < 0.001; NS: not significant. GraphPad PRISM 5.0 was used for generating the Kaplan–Meier curves and the graphs.

## 3. Results

### 3.1. Transcriptional Profiling of Fxr^ex5∆^ Hepatocytes

**Figure 1:**
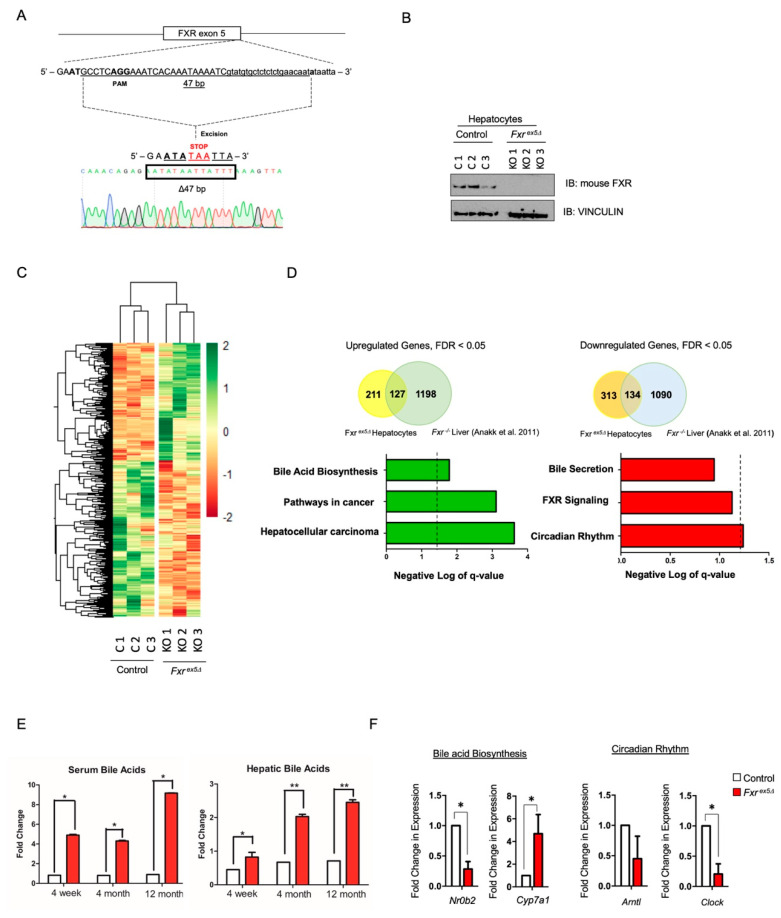
Generation of CRISPR knockout FXR mice. (**A**) Single cell embryos were injected with guide RNA that targets exon 5 of *Fxr*. The embryos were implanted into pseudo-pregnant females to generate founders. The sequencing results show that the mutation was a 47 base-pair deletion at exon 5. (**B**) Hepatocytes were isolated from control and *Fxr^ex5Δ^* livers using the classic collagenase-perfusion method. Western blot was performed on whole cell lysates from control and *Fxr^ex5Δ^* hepatocytes. VINCULIN was used as a loading control. (**C**) RNA-seq was performed on control and *Fxr ^ex5Δ^* hepatocytes. Heatmap of 789 differentially expressed genes between control and *Fxr^ex5Δ^* hepatocytes (*p* value and FDR value cut-off of 0.05). (**D**) Overlap of dysregulated genes from *Fxr^ex5Δ^* hepatocyte RNAseq with *Fxr^ex5Δ^* liver RNAseq obtained from Annak et al. (2011) [22]. Pathways analysis of all upregulated and downregulated genes was conducted using the KEGG and REACTOME databases. The cut-off for pathways analysis was chosen as *p*-value < 0.05, FDR < 0.1. The dotted line represents a *FDR* value of 0.05. Any value on the right of the dotted line signifies pathways that pass the *FDR* threshold of 0.05. (**E**) Hepatic and serum bile acid analysis at 4-week, 4-month and 12-month time-points in control and *Fxr^ex5Δ^* animals (*n* = 5). (**F**) Expression analysis of selected downregulated and upregulated genes upon FXR deletion. The normalized FPKM values from RNAseq analysis were averaged for control and *Fxr^ex5Δ^* hepatocytes and fold-changes were calculated (*n* = 3). T-test was performed to determine statistical significance relative to controls: * *p* < 0.05, ** *p* < 0.01.

The FXR knockout mice were generated by targeting exon 5 of the full-length FXR protein, which encodes part of the hinge domain and the ligand-binding domain (Figure 1A, Figure S1A) [25]. Mutant *Fxr* exon 5 alleles were sequenced and a 47 bp deletion was identified resulting in a frame-shift mutation (Figure 1A, Figure S1B–D). This frame-shift mutation generated a premature stop codon at residue 281 of the full-length FXR protein (Figure S1E). Western blot analysis using a pan-FXR antibody (recognizes residues 2-126 of FXR) on liver samples from wildtype, *Fxr* heterozygous and homozygous animals confirmed loss of FXR expression in the liver (Figure S1F). Aging studies demonstrated that at approximately 8 months post birth, the *Fxr^ex5∆^* animals develop liver abnormalities including neoplastic foci of hepatocellular carcinoma (HCC) between 8 and 15 months of age, relative to age and sex-matched control animals (Gehan-Breslow-Wilcoxon test *p* < 0.001) (Figure S1G). Consistent with previous studies [26], there was a lack of sex-specific dimorphism in the onset of HCC in *Fxr^ex5∆^* animals. Morphologically, the *Fxr^ex5∆^* livers displayed an irregular and grainy surface with multiple HCC foci of varying dimensions. Hematoxylin and eosin staining of livers from *Fxr^ex5∆^* animals showed macro and micro vesicular steatosis and inflammation in the liver (Figure S1H). These observations support the presence of HCC in *Fxr^ex5∆^* animals.

The liver is mostly comprised of parenchymal cells consisting of hepatocytes that occupy approximately 80% of the liver tissue [27]. Although transcriptional changes upon FXR deletion in whole liver have been reported [22], expression changes in hepatocytes remain unclear. Therefore, hepatocytes were isolated from control and *Fxr^ex5∆^* livers using the classic collagenase-perfusion technique with the addition of a percoll density gradient to increase the purity and viability of hepatocytes [23]. Western blot analysis demonstrated a complete loss of FXR in *Fxr^ex5∆^* hepatocytes (Figure 1B). To determine the transcriptional changes associated with FXR deletion, RNA sequencing (RNAseq) was performed on age and sex matched control and *Fxr^ex5∆^* hepatocytes (Figure 1C and Figure S2A–C, Supplementary Table S1). Hierarchical clustering demonstrated clear differences between control and *Fxr^ex5∆^* hepatocytes (Figure 1C). We identified 447 downregulated (~57%) and 338 upregulated (43%) genes upon FXR deletion using a *p*-value and FDR cut-off of <0.05. Interestingly, a significant portion of the dysregulated genes in *Fxr^ex5∆^* hepatocytes were also found to be dysregulated in FXR^−/−^ liver [22] (Figure 1D). Pathway analysis of upregulated and downregulated genes identified FXR signaling and bile acid biosynthesis as a dysregulated pathway, respectively (Figure 1D). Consistent with this analysis, upregulation of the CYP7A1 protein, the rate limiting enzyme in bile acid biosynthesis, was observed in *Fxr^ex5∆^* animals, as well as significant increases in serum and hepatic bile acid levels (Figure 1E and Figure S2D). Pathway analysis also identified circadian rhythm as highly dysregulated pathways, which are independent risk factors for hepatocarcinogenesis and have been previously linked to FXR signalling [28,29]. Consistent with these observations, downregulation of genes relevant to the bile acid biosynthesis and circadian signaling pathways were observed in hepatocytes upon FXR deletion (Figure 1F). These analyses suggest that a loss of FXR leads to HCC potentially via a combined dysregulation of the bile acid biosynthesis and circadian rhythm signaling pathways.

### 3.2. Loss of Chromatin Accessibility in Fxr^ex5∆^ Hepatocytes

**Figure 2:**
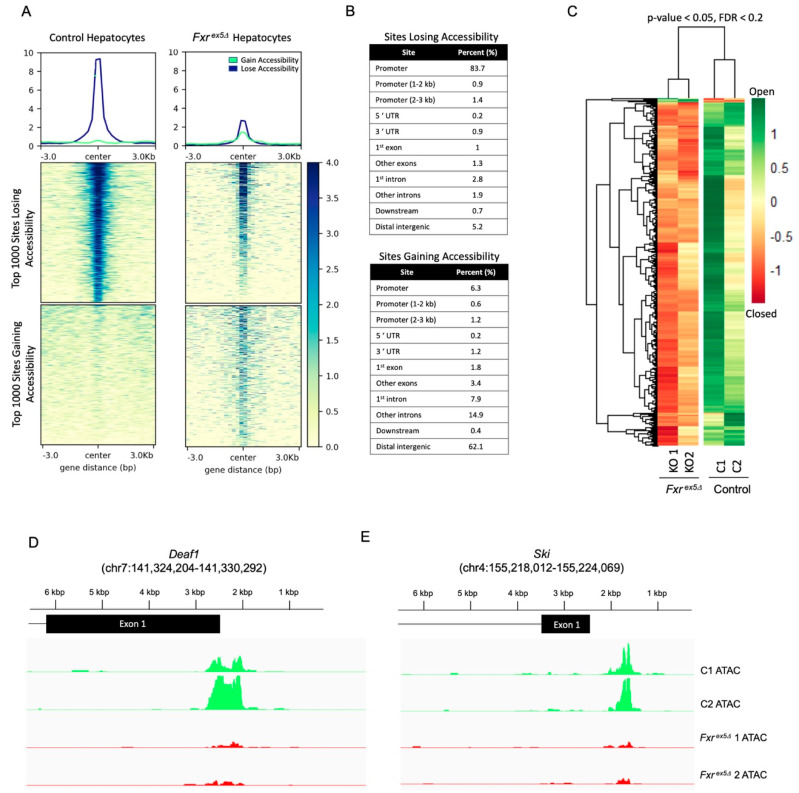
Global changes in chromatin accessibility in *Fxr^ex5Δ^* hepatocytes. (**A**) ATAC-seq was performed on control and *Fxr^ex5Δ^* hepatocytes. Heatmap of 1592 differentially accessible regions that pass the FDR cut-off of 0.2. (**B**) Table showing distribution of annotated genomic features for the top 1000 peaks with loss or gain of accessibility with *Fxr^ex5Δ^* in hepatocytes. (**C**) Top 1000 differentially accessible normalized ATAC-Seq peaks were separated by loss or gain of accessibility. Then, heatmap of signal distribution around ATAC-Seq peak summits was generated for both control and *Fxr^ex5Δ^* samples. (**D**,**E**) Genomic tracts of ATAC-seq signal in control and *Fxr^ex5Δ^* hepatocytes at the top two differentially accessible promoters, *Deaf1* (**D**) and *Ski* (**E**) genes.

To assess changes in chromatin accessibility, we performed ATAC-seq on the same control and *Fxr^ex5∆^* hepatocytes that were used for transcriptional profiling. Differential accessibility analysis identified a total of 73,670 regions losing or gaining accessibility between the control and *Fxr^ex5∆^* hepatocytes. The top 1000 peaks with increased or decreased accessibility were then shortlisted to detect overall patterns in chromatin accessibility (Figure 2A, Supplementary Tables S2 and S3). Genomic sites that gained the most accessibility in *Fxr^ex5∆^* hepatocytes were already open in control samples and became even more accessible in *Fxr^ex5∆^* hepatocytes. In contrast, genomic sites that lost the most accessibility in *Fxr^ex5∆^* hepatocytes were largely accessible in control samples. Consistent with these findings, a significant reduction in overlap between ATAC peaks and H3K4me3/ H3K27ac ChIP-seq, which are markers of open chromatin, was observed upon the loss of FXR (Figure S3B). Interestingly, the majority of peaks that lost accessibility in *Fxr^ex5∆^* hepatocytes were located in the promoters of specific genes approximately 1 to 3 Kb from the transcription start site (TSS), whereas regions that gained accessibility upon the loss of FXR were found in the distal intergenic and intronic regions of the genome (Figure 2B). Interestingly, none of the sites that are gaining accessibility were associated with promoted genes that are involved in the regulation of chromatin accessibility. Annotation of the FXR binding sites using a publicly available ChIP-seq dataset demonstrates that FXR predominantly occupies the promoter or distal intergenic regions in chromatin (Figure S3C). Therefore, these findings broadly suggest that the deletion of FXR primarily leads to a loss in direct promoter activity of target genes, and an increase in enhancer activity in a subset of genes in hepatocytes.

From the 73,670 peaks undergoing a change in accessibility with FXR deletion, 1592 peaks met the FDR cut-off of 0.2 (Figure 2C, Figure S3A). Hierarchical clustering of the FDR adjusted peaks demonstrate clear differences between control and *Fxr^ex5∆^*, with most of the differential peaks corresponding to regions of chromatin becoming inaccessible in *Fxr^ex5∆^.* Surprisingly, of the 1592 peaks that met the FDR cut-off, only 1.3% (21 peaks) of the 1592 FDR adjusted peaks correspond to chromatin regions that gain accessibility in *Fxr^ex5∆^* hepatocytes. The most dramatic loss in chromatin accessibility in *Fxr^ex5∆^* hepatocytes was found at the promoters of *Deaf1* (Figure 2D) and *Ski* transcription factors (Figure 2E). Collectively, these findings demonstrate that the chromatin is largely undergoing a loss of accessibility upon FXR deletion in hepatocytes.

### 3.3. FXR Deletion Affects Promoter Activity and Transcription Factor Binding at Target Genes

**Figure 3:**
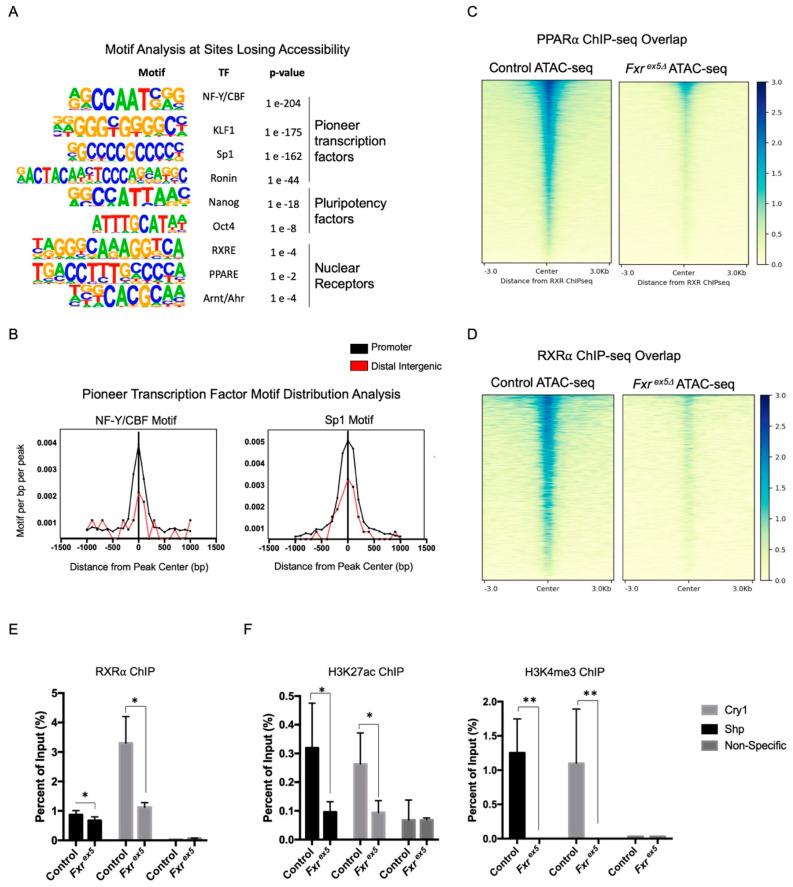
Loss of FXR affects chromatin accessibility at transcription factor binding sites and the recruitment of transcription factors. (**A**) Motif enrichment analysis of top sites losing accessibility upon FXR deletion. (**B**) Motif distribution analysis on promoter or distal intergenic regions for peaks losing accessibility with FXR deletion. (**C**,**D**) Overlap in chromatin accessibility in control and *Fxr^ex5Δ^* ATAC-seq and ChIP-seq peaks of transcription factors PPARα (**C**) and RXRa (**D**) The ATAC-seq signal intensity heatmap depicts profile of transcription factor binding at sites of chromatin accessibility in control and *Fxr^ex5∆^* hepatocytes. (**E**,**F**) ChIP at the promoters of *Nr0b2* genes using antibodies specific for RXRa (**E**), H3K27 acetylation or H3K4 trimethylation (**F**) in control and *Fxr^ex5∆^* hepatocytes. A control region in the *Hic1* locus was used as a negative control for the test. All ChIP experiments were performed using hepatocytes from three separate male animals (*n* = 3). Student T-test was performed in order to determine statistical significance relative to controls: * *p* < 0.05, ** *p* < 0.01.

The observation that chromatin loses accessibility with FXR deletion prompted us to investigate if access to transcription factor binding is reduced in *Fxr^ex5∆^* hepatocytes. Therefore, motif enrichment analysis was performed for known motifs on the differentially accessible regions (FDR < 0.2). Motif analysis on regions gaining accessibility failed to generate any statistically significant motifs. In contrast, 159 motifs were enriched in peaks that lose accessibility with FXR deletion in hepatocytes (*p*-value < 0.05, q-value < 0.05) (Figure 3A). Consistent with this observation, motifs that are predicted to bind NF-Y/CBF, as well as members of Sp1 and Krüppel-like factor (*Klf*) family of transcription factors were highly enriched in regions that become inaccessible upon FXR deletion (Figure 3A). NF-Y is a heterotrimeric transcription factor that binds the CCAAT box element in the promoter, and recruits RNA polymerase II to activate transcription [30]. The SP1/KLF family of transcription factors are essential components of the eukaryotic transcriptional machinery [31]. Both NF-Y and SP1/KLF transcription factors are essential for promoter activity via the recruitment of co-activators such as CBP/p300 as well as members of the core transcriptional machinery. Importantly, NF-Y has been shown to maintain regions upstream of the TSS in a nucleosome depleted state [32], and the post-natal inactivation of NF-Y has been linked to hepatocellular carcinoma [33]. In agreement with the promoter binding activity of SP1/KLF and NF-Y transcription factors, their respective motifs were most enriched in the promoter annotated peaks that are losing accessibility with FXR deletion (Figure 3B). Combined with the observation that the expression of NF-Y and SP1/FKL transcription factors do not change between groups, these findings strongly suggest that the deletion of FXR leads to a loss of promoter activity at target genes.

The motifs for pluripotency factors, such as Nanog and Oct4, were also enriched in sites losing accessibility with FXR deletion. This agrees with a recent finding that FXR regulates stem cell proliferation [34]. Among the nuclear receptor motifs which are enriched in regions losing accessibility in *Fxr^ex5∆^* hepatocytes is the DR1 motif, a consensus motif for PPAR/RXR heterodimers (Figure S4A). Consistent with this observation, a significant reduction in the overlap of RXR_α_ and PPAR_α_ ChIP-seq with *Fxr^ex5∆^* ATAC peaks was found, relative to the controls (Figure 3C,D). Since RXR_α_ heterodimerizes with FXR_α_ to regulate gene transcription, RXR_α_ ChIP was performed at the promoters of *Nr0b2* and *Cry1* FXR target genes. ChIP was also performed using antibodies specific to the H3K27 acetylation (H3K27ac) and H3K4 trimethylation (H3K4me3) activating histone modifications, which are markers of open chromatin. Surprisingly, upon FXR deletion loss of RXR_α_ recruitment, as well as H3K27ac and H3K4me3 activating histone marks was found at *Nr0b2* and *Cry1* promoters (Figure 3E,F). Collectively, these findings strongly suggest that the deletion of FXR leads to the reduction in accessibility to transcription factor motifs and transcription factor binding sites at target genes.

### 3.4. FXR Deletion Leads to Dysregulation of the Bile Acid and Circadian Rhythm Pathways

**Figure 4:**
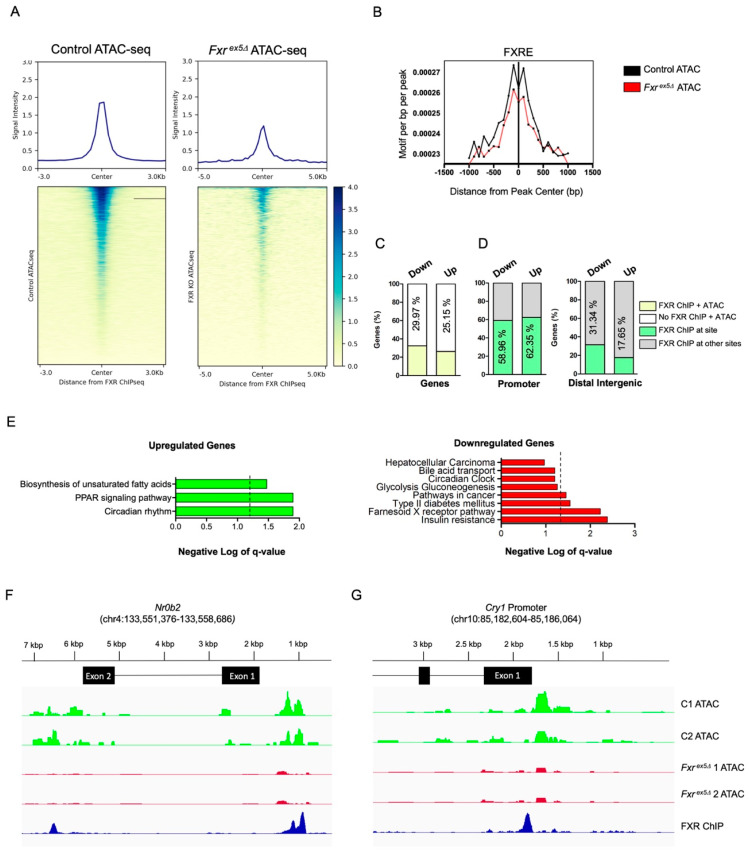
FXR deletion leads to loss of chromatin accessibility and transcriptional dysregulation of genes from the circadian and insulin pathways. (**A**) The FXR ChIP-seq peak tracks were overlapped with ATAC-seq peaks from control and *Fxr^ex5Δ^* samples. The heatmap depicts intensity of FXR binding at sites of chromatin accessibility. (**B**) Motif de-enrichment plot for the FXR consensus motif for control and *Fxr^ex5Δ^* hepatocytes. (**C**,**D**) Overlap between downregulated (447) and upregulated (338) genes that have an FXR binding site and are undergoing changes in chromatin accessibility upon FXR deletion in hepatocytes. Annotation of FXR ChIP-seq peak summits at genes that are both dysregulated upon FXR deletion and with changes in chromatin accessibility (**D**). (**E**) Pathway analysis of upregulated and downregulated genes that are both bound by FXR and undergoing changes in chromatin accessibility. The dotted line represents a *FDR* value of 0.05. Any value on the right of the dotted line signifies pathways that pass the *FDR* threshold of 0.05. (**F**,**G**) Browser view of the *Nr0b2* (**F**) and *Cry1* (**G**) loci showing control and *Fxr^ex5∆^* ATAC-seq normalized tracks alongside of FXR ChIP-seq peaks. These genes are relevant to the circadian rhythm, bile acid biosynthesis and insulin signaling pathways, respectively.

The deletion of FXR causes a global loss of accessibility at specific transcription factor binding motifs, which correlated with a reduction in their binding sites in *Fxr^ex5∆^* hepatocytes. In concert with these findings, the binding sites of FXR, and access to its IR1 motif, were found to be reduced in *Fxr^ex5∆^* hepatocytes (Figure 4A,B). CBP and its orthologue p300 are essential coactivators that are recruited by FXR [20]. We sought to determine if there is a correlation between FXR deletion and binding of CBP/p300 at FXR target genes. A significant reduction in the CBP/p300 co-activator binding sites was observed upon FXR deletion in *Fxr^ex5∆^* hepatocytes (Figure S4C). In addition, an enrichment of CTCF binding was observed in sites losing accessibility in *Fxr^ex5∆^* hepatocytes, which further strengthens the finding that loss of FXR alters chromatin architecture (Figure S4E). An overlap of FXR ChIPseq with ChIPseq of CBP, CTCF, and members of the pioneer family (KLF6 and FOXA1) of transcription factors demonstrated that the binding of these proteins is centered around FXR recruitment (Figure S5). This finding indicates that FXR potentially regulates regulate chromatin architecture via the recruitment of CBP, CTCF, KLF6 and CTCF transcription factors. Combined with the finding that the majority of transcriptional changes associated with FXR deletion correspond to downregulated genes, these observations strongly suggests that loss of FXR contributes to transcriptional silencing via reduction in accessibility to transcription factors and loss of recruitment of co-activators. 

We found that 29.8% of the genes dysregulated in *Fxr^ex5∆^* hepatocytes (234 of 785 genes) also have FXR binding sites. Interestingly, the majority of FXR binding was found 1–3 kb from the TSS, which indicates that FXR plays a critical role in regulating promoter-specific activity at target genes. Further analysis indicated that approximately 32.44% of downregulated and 26.33% of upregulated genes in *Fxr^ex5∆^* hepatocytes contain FXR binding sites (Supplementary Figure S4B). Of the 234 genes dysregulated in *Fxr^ex5∆^* hepatocytes, 124 (52.99%) have binding sites in the promoter and 44 genes (18.8%) have binding sites for FXR in the distal intergenic regions. However, only 17 genes (or 7.2%) of the 234 dysregulated genes possessed an FXR binding site at both the promoter and distal intergenic regions. These findings agree with the observation that the majority of the FXR binding events are localized within the promoter or distal intergenic regions of target genes (Figure S3C). To determine if changes in accessibility correlate with altered gene expression, we compared dysregulated genes containing an FXR binding site and ATACseq peaks. Surprisingly, majority of the dysregulated genes known to be bound by FXR were also found to have changes in accessibility upon FXR deletion (Figure 4B). In agreement with previous analysis, changes in chromatin accessibility at these genes were predominantly localized to the promoter or distal intergenic regions (Figure 4C). Pathways analysis of the downregulated genes using the KEGG and REACTOME databases identified FXR signaling as one of the top dysregulated pathways (Figure 4D). In addition, bile acid and the circadian rhythms pathways, as well as genes implicated in HCC were identified using pathways analysis. This is in concert with our previous analysis on the transcriptional changes between control and *Fxr^ex5∆^* hepatocytes. In agreement with the pathways analysis, FXR was found to localize at *Nr0b2* and *Cry1* genes that play an important role in bile acid and circadian homeostasis, respectively (Figure 4E,F). These genes were also found to be downregulated and lose chromatin accessibility upon FXR deletion in hepatocytes (Figure 4E,F, Figure S4D). Collectively, these findings provide strong evidence that a loss of FXR potentially predisposes mice to HCC by the combined transcriptional and chromatin dysregulation at genes important for the maintenance of BA and circadian homeostasis in hepatocytes.

## 4. Discussion

FXR is a member of the nuclear receptor superfamily and plays an important role in the transcriptional regulation of genes essential for the synthesis, transport and detoxification of BA. Although the loss of FXR has been studied in the context of whole liver, the implications of FXR deletion in hepatocytes is unclear. Hepatocytes are the main functional parenchymal cells in the liver, responsible for the majority of its metabolic activity, and comprise approximately 80% of the total liver mass. In this study, we isolated hepatocytes from the control and *Fxr^ex5∆^* animals and examined the transcriptional dysregulation, as well as alterations in chromatin accessibility resulting from a loss of FXR. The isolation of hepatocytes involved a two-step perfusion and collagenase digestion, followed by a low centrifugal spin which separates hepatocytes from other cell types, such as Kupffer and sinusoidal endothelial cells. Based on our analysis, 29.8% of the genes that were dysregulated upon the loss of FXR also possess binding sites for FXR. This indicates that the changes in expression involve multiple mechanisms in addition to direct regulation by FXR binding. In addition, a subset of FXR binding was also found at distal intergenic regions of genes, which provides evidence of a potential role of FXR in regulating cis-regulatory elements to modulate transcription. Enhancers are a class of cis-regulatory element that interact with promoter of neighbouring genes, via a three dimensional looping of the chromatin, in order to regulate their transcription [35]. Although nearly a quarter of total FXR binding sites are localized to the distal intergenic regions, the role of FXR as a cis-acting transcription factor and in the regulation of enhancer activity remains unclear.

Unlike the forkhead pioneering transcription factors, such as FOXA2, that bind condensed chromatin and recruit additional transcription factors to increase accessibility, the changes in chromatin accessibility associated with a loss of FXR were not dramatic. For example, we did not observe a complete gain or loss of chromatin accessibility with FXR deletion at any of the differentially accessible regions. However, the changes in chromatin accessibility observed with FXR deletion were more pronounced relative to a loss of other nuclear receptors in the liver, such as LXR [36]. For instance, upon FXR deletion the vast majority (>95%) of statistically significant differentially accessible regions correspond to chromatin that is closing relative to the same regions in control hepatocytes. The deletion of FXR also corresponded with a dramatic loss of accessibility at FXR binding sites, as well as the binding sites of other nuclear receptors, transcription factors, and the transcriptional co-activator CBP. The majority of sites losing accessibility were found to be embedded in the promoter region of target genes. This is in agreement with the observation that FXR binds predominantly at the promoter region of genes that are dysregulated in *Fxr^ex5∆^* hepatocytes. 

Importantly, this finding correlated with the promoter-specific loss of accessibility of motifs for NFY/CBF and SP1/KLF family of pioneer transcription factors that are important for promoter activity and accessibility. Additionally, regions losing accessibility upon FXR deletion were enriched for the motif for CTCF, which is essential for mediating accessibility at genes essential for somatic cell reprogramming [37]. This indicates a potential involvement of FXR in cellular differentiation and pluripotency. Increases in chromatin accessibility with the loss of FXR were mostly localized to the distal intergenic regions, which might be indicative of compensatory mechanisms to overcome the loss of promoter activity at target genes. Interestingly a major binding site that was identified in regions becoming inaccessible in *Fxr^ex5∆^* hepatocytes was the DR1 motif which binds PPAR/RXR. Previous studies have shown that FXR and PPAR_α_ possess interdependent functional roles in the liver with respect to energy balance and our analysis would seem to confirm this observation [38].

An overlap of the RNA-seq and ATAC-seq dataset identified dysregulation in FXR signaling and bile acid homeostasis, as well as the onset of hepatocellular carcinoma which is in agreement with previous studies [22,24,26]. Interestingly, pathways analysis also identified genes that are essential for the circadian and BA homeostasis in the liver. Importantly, FXR was found to localize at the promoter of these genes, and the loss of FXR led to a decrease in chromatin accessibility. These findings indicate that FXR is involved in mediating chromatin accessibility at the promoters of target genes which is essential for their transcription. This is in agreement with previous findings which demonstrate that FXR recruits the pioneering transcription factor Foxa2 [39], as well as CBP [20] to increase chromatin accessibility at target genes. Other pathways relevant to insulin and circadian signaling were also identified, such as an onset of type 2 diabetes and circadian clock. FXR has been previously linked to play an essential role in maintaining glucose and bile acid homeostasis, which are both independent risk factor for HCC [29,40,41]. In humans, a loss of function mutation in the *FXR* gene has recently been linked to a severe autosomal recessive liver disorder classified as progressive familial intrahepatic cholestasis 5 (PFIC5), which increases the risk of HCC development [42,43,44]. The loss of FXR has also been shown to accelerate the onset of HCC resulting from disruption in circadian rhythm in mice, which indicates that it functions as a regulator of circadian homeostasis in the liver [28].

## 5. Conclusions

Overall, these findings indicate that the loss of FXR causes HCC from a combined dysregulation in the bile acid, circadian and insulin signaling pathways via a loss of promoter accessibility and activity at genes relevant to these pathways.

## Figures and Tables

**Table 1:** sgRNA sequence used for CRISPR/Cas9 to generate *Fxr^ex5∆^* animals.

sgRNA	Sequence (5′–3′)
FXR sgRNA	TTCTAATACGACTCACTATAGCAACAAACAGAGAATGCCTCGTTTTAGAGCTAGA

**Table 2:** Primers for genotyping.

Primer	Sequence (5′–3′)
FXR forward	ATATGCCTTTGACCGCCCTC
FXR reverse	GGCACACTTTACATATTTCAAGAAC
FXR reverse (47 bp deletion)	CACATTTACATATAAATCCCACC

**Table 3:** Primers for RT-PCR and ChIP-qPCR.

Primer	Forward (5′–3′)	Reverse (5′–3′)
FXR exon 1	GTGTGAAGCCAGCTAAAGGTATGC	TGTGGCTGAACTTGAGGAAACGG
FXR exon 5	GCTGATCAGACAGCTAATGAGG	GTGATTTCCTGAGGCATTC
FXR exon 9	CCTCTCTCCAGACAGAC	GGTTCTCAGGCTGGTACATCTTGC
Non-specific Region (130 bp from *Hic1* TSS)	TCTTGCTCCCGTCTTCCTTA	CATTCAGGGCCGAGAAGTT
Cry1 Promoter (132 bp from *Cry1* TSS)	GGAGCAGAACTATGCCTCCTC	GACCGGTTGCGATCGCTG
Nr0b2 Promoter (247 bp from *Nr0b2* TSS)	GCCTGAGACCTTGGTGCCCTG	CTGCCCACTGCCTGGATGC

**Table 4:** List of reagents.

Reagent	Source	Identifier
FXR	R&D system	Cat#: PP-A9033A-00
CYP7A1	Abcam	Cat#: Ab65596
VINCULIN	Sigma	Cat#: V9264
Percoll	Santa Cruz	Cat#: sc-500790A
Total Bile Acid Assay Kit	Diazyme	Cat#: DZ042A-K01
RNAzol	Sigma	Cat#: R4533-50ML
Collagenase	Worthington	Cat#: LK002066
Avertin	Sigma	Cat#: T48402
T-amyl alcohol	Sigma	Cat#:240486
HBSS	Wisent	Cat#: 311-512-CL

## Data Availability

The RNA-seq dataset of *Fxr^ex5Δ^* liver was acquired from The Signaling Pathways Project (signalingpathways.org).

## Supplementary Materials

The following supporting information can be downloaded at: https://www.mdpi.com/article/10.3390/cancers14246191/s1, Table S1: Enriched motifs in promoters; Table S2: Differential accessibility peaks; Table S3: Differentially expressed genes. Figure S1: Generation of the FXR knockout mice using CRISPR and aging studies; Figure S2: Validation of the RNA-seq. (A) The top most downregulated genes were validated using RT-qPCR (*n* = 3); Figre S3: FXR ChIPseq annotation, motif, and ATAC seq analysis; Figure S4: Motif distribution analysis and ChIPseq overlaps; Figure S5: Overlap of FXR ChIPseq with transcription factors CBP, KLF6, CTCF, and FOXA1.
