# Supplementary material for: Regulation of Chromatin Accessibility by the Farnesoid X Receptor Is Essential for Circadian and Bile Acid Homeostasis In Vivo
Source: Cancers (Basel). 2022 Dec 15;14(24):6191. doi: 10.3390/cancers14246191 (PMC9777377; doi:10.3390/cancers14246191)
Supplement: Supplementary file 1 [file cancers-14-06191-s001.zip › cancers-1891213-supplementary.pdf]

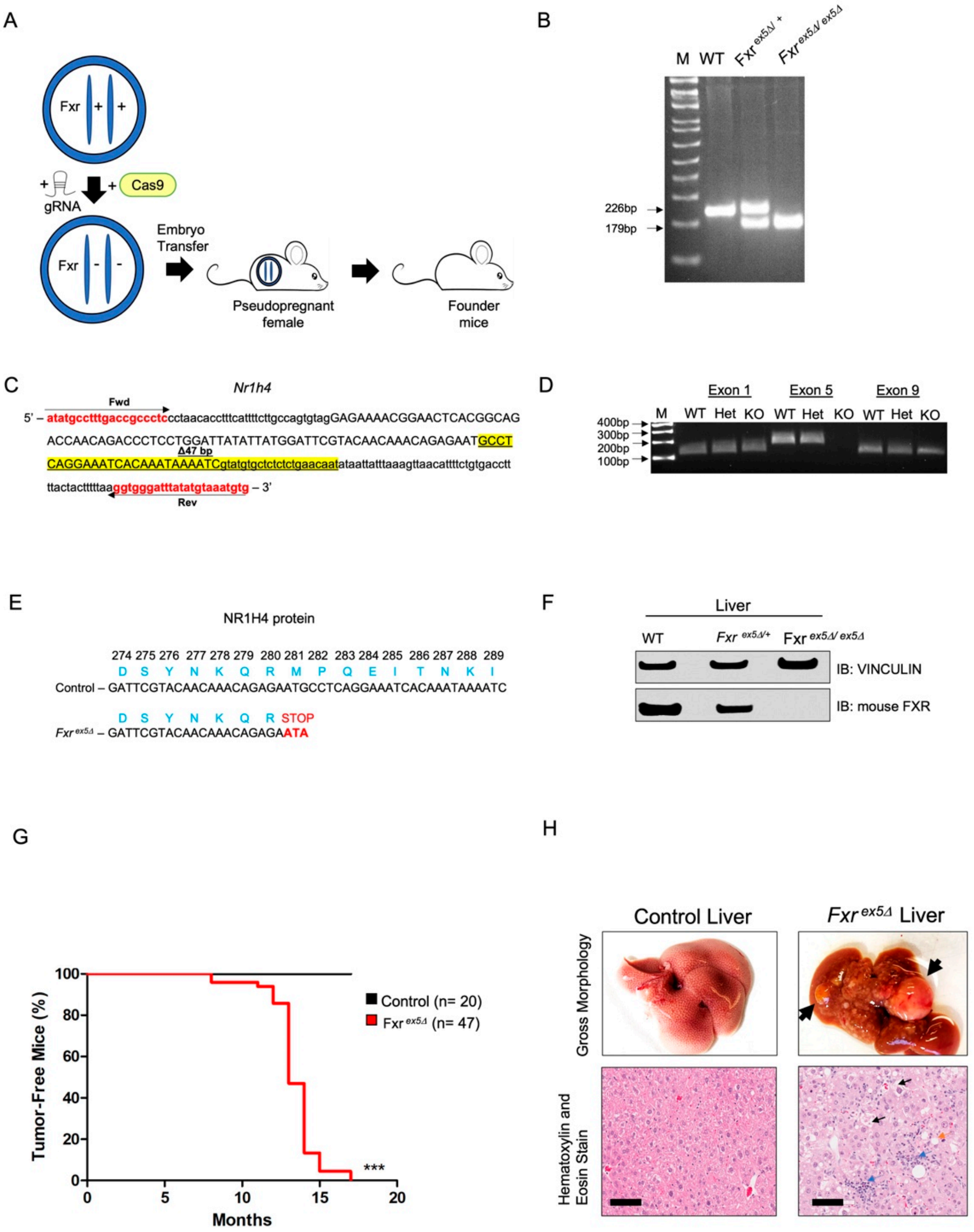

Figure S1. Generation of the FXR knockout mice using CRISPR and aging studies. (**A**) Experimental design for Fxr deletion in mice using CRISPR. Single cell embryos were injected with guide RNA that targets exon 5 of Fxr, then the embryos were implanted into pseudo-pregnant females to generate founders. (**B**) Genotyping for wildtype mice, which only have the WT allele, heterozygous mice which have both alleles, and knockout mice which only have the knockout allele incorporating a 47 bp deletion. (**C**) Schematic depicting where genotyping primers bind relative to 47 bp deletion. (**D**) RT-PCR experiment confirming that the deletion took place within exon 5, you can see that the wildtype and the heterozygotes produce have the amplicon at exon 5 but the knockouts do not. (**E**) Translation of interrupted CDS sequence in $Fxr^{ex5\Delta}$ locus (**F**) Immunoblot (IB) of FXR in *Wt*, *Fxr* heterozygous and *Fxr* knockout mice livers. VINCULIN was used as a loading control. (**G**) Kaplan–Meier curve demonstrating high penetrance of liver cancer upon whole body FXR deletion. (**H**) Representative liver morphology and H&E staining of end point control and $Fxr^{ex5\Delta}$ animals. The black arrows indicate foci of HCC in low field microscopy. The black arrows in high field microscopy indicate micro-vesicular steatosis, whereas the orange arrows indicate macro-vesicular

steatosis. The blue arrows indicate the presence of inflammatory cells. In contrast, the control liver sections demonstrate an absence of steatosis. Scale bars in high-power field images represent 100 µm. Gehan-Breslow-Wilcoxon test p test was performed in order to determine statistical significance relative to controls: * $p < 0.05$, ** $p < 0.01$, *** $p < 0.001$.

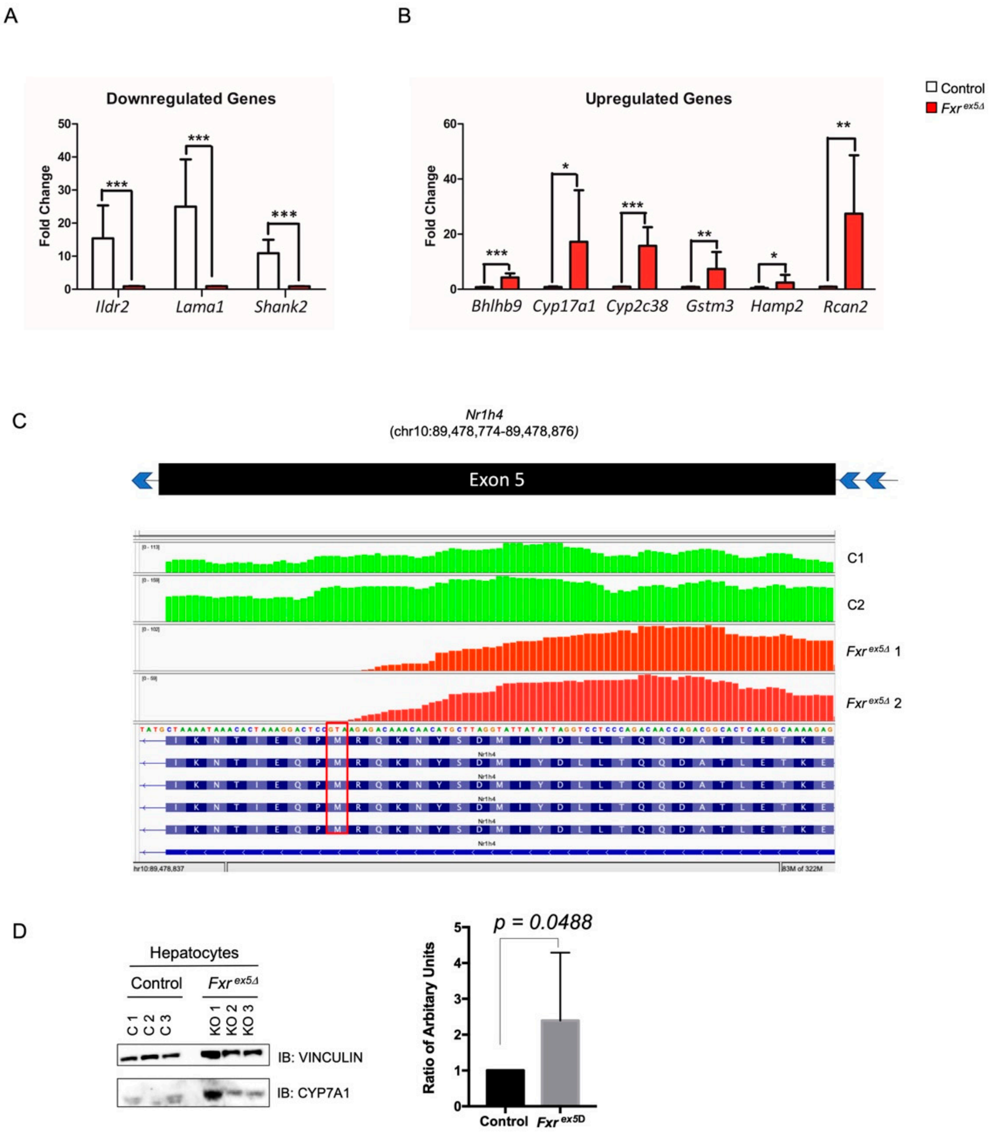

Figure S2. Validation of the RNA-seq. (**A**) The top most downregulated genes were validated using RT-qPCR (n = 3). (**B**) The top most upregulated genes were validated using RT-qPCR (n = 3). (**C**) IGV plot of exon 5 of FXR demonstrating loss of expression signal at the mutant allele in $Fxr^{ex5\Delta}$ hepatocytes. The red box indicates residue 281 where the premature stop codon is formed (**D**) Validation of the CYP7A1 upregulation using a Western blot. The Western blot was then analyzed by imageJ to determine fold change in protein.

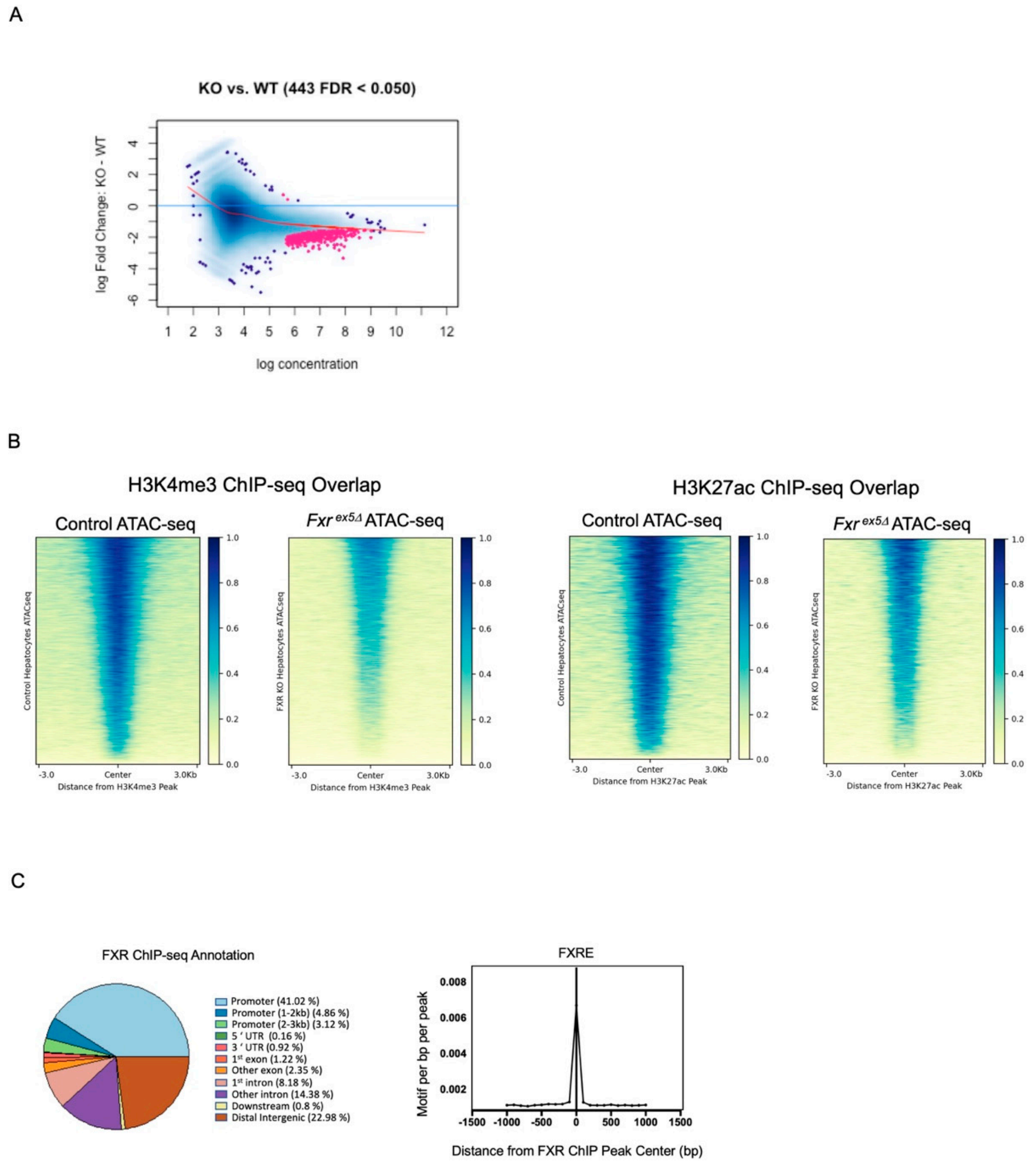

Figure S3. FXR ChIPseq annotation, motif, and ATAC seq analysis. (**A**) MA plot for ATAC seq analysis. The highlighted points in MA plot correspond to ATAC peaks that pass the FDR < 0.05. (**B**) Heatmap depicting an overlap of the H3K27ac and h3k4me3 ChIP-seq peaks with control and *Fxr* $^{ex5\Delta}$ ATAC-seq data. (**C**) FXR ChIP-seq annotation and FXRE distribution around FXR ChIP-seq tracks.

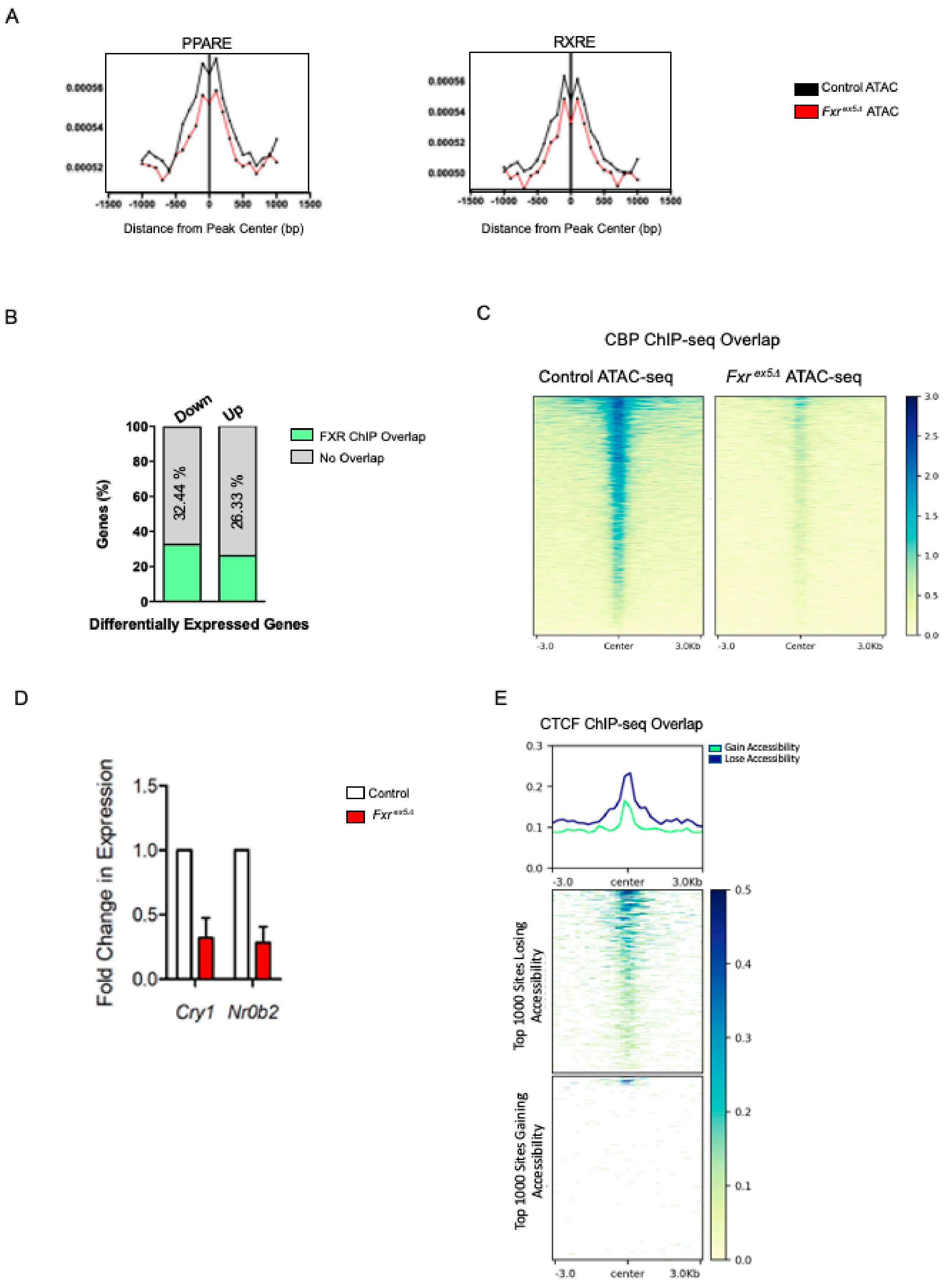

Figure S4. Motif distribution analysis and ChIPseq overlaps (**A**) Transcription factor motif distribution around ATAC-Seq peaks of control and *Fxr*$^{ex5\Delta}$ samples. (**B**) Publicly available FXR ChIP-seq dataset was used to determine FXR binding at downregulated (n = 447) and upregulated (n = 338) genes upon FXR deletion. **(C)** Overlap between chromatin accessibility in control and *Fxr*$^{ex5\Delta}$ ATAC-seq and ChIP-seq peak summits of the transcriptional co-activator CBP/ p300. (**D**) The transcriptional changes in *Cry1* and *Nrob2* between control *Fxr*$^{ex5\Delta}$ hepatocytes was analyzed by FPKM values generated through RNA-seq (n = 3). (**E**) Overlap between CTCF ChIP-seq peak summits and regions that are closing or opening upon FXR deletion in *Fxr*$^{ex5\Delta}$ hepatocytes.

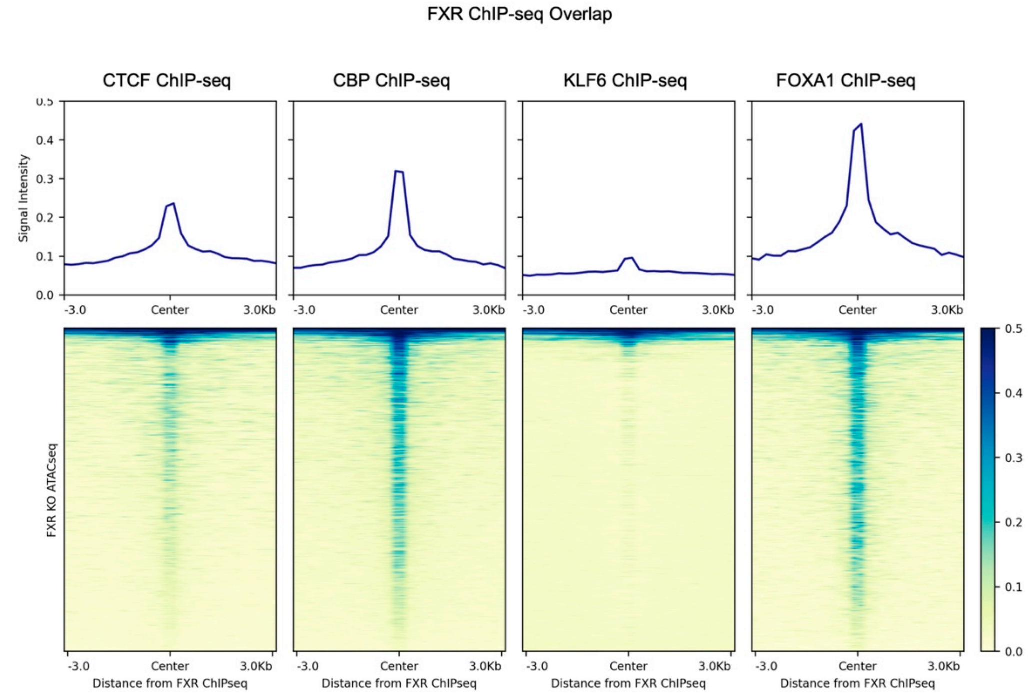

Figure S5. Overlap of FXR ChIPseq with transcription factors CBP, KLF6, CTCF, and FOXA1. The overlap of FXR ChIPseq with pioneer transcription factors KLF6 and FOXA1, as well, transcription factors CBP and CTCF was performed using Deeptools.
